# Disease control, psychiatric comorbidity, health-related quality of life, and experiences of care during transition to adult healthcare: a single-center prospective study

**DOI:** 10.1007/s00431-026-06791-z

**Published:** 2026-03-11

**Authors:** Mira Kallio, Anna Alanen, Kaija-Leena Kolho, Heikki Relas, Silja Kosola

**Affiliations:** 1 Department of Pediatrics, University of Helsinki and Helsinki University Hospital, Helsinki, Finland; 2 New Children`s Hospital, Pediatric Research Center, University of Helsinki and Helsinki University Hospital, Helsinki, Finland; 3 Department of Nursing Science, University of Turku, Turku, Finland; 4 Inflammation Center, University of Helsinki and Helsinki University Hospital, Helsinki, Finland

**Keywords:** Transfer of care, Chronic conditions, Adolescents, Young adults

## Abstract

This work aims to study the changes in disease control during the transition to adult healthcare and the associations of disease control and psychiatric comorbidity with health-related quality of life (HRQoL) and experiences of care. Participants were adolescents and young adults (AYAs) with various chronic conditions, recruited from the New Children’s Hospital in Helsinki, Finland. Data were collected before and 1 and 2 years after the transfer of care. Disease control was measured by predefined criteria and categorized as good, moderate, or poor. Psychiatric diagnoses were obtained from medical records. HRQoL was assessed using the Pediatric Quality of Life Inventory and the 16D instruments, while experiences of care were evaluated with questions from the Adolescent Friendly Hospital Survey. Of the 253 AYAs, 199 (79%) and 172 (68%) completed surveys 1 and 2 years after the transfer of care. The mean ages were 17.2, 18.3, and 19.8 years, respectively. Disease control was determined for 251 (99%) AYAs before transfer, 206 (81%) 1 year after, and 208 (82%) 2 years after the transfer of care. Disease control remained stable in most AYAs after transfer (good disease control: *n* = 70 (28%) before transfer, *n* = 94 (46%) 1 year, and *n* = 87 (42%) 2 years after the transfer of care) and showed no association with HRQoL or experiences of care. AYAs without psychiatric comorbidity had better disease control and HRQoL compared to those with a psychiatric diagnosis.

*Conclusion*: Disease control in AYAs with chronic conditions remained stable throughout the transition to adult healthcare. Psychiatric comorbidity is an important contributor to inferior disease control and HRQoL across the transfer of care.

*Trial registration*: Registration name, “The Bridge”; registration number, NCT04631965 (https://clinicaltrials.gov/ct2/show/NCT04631965).

**Table:** 

**What is Known:** *• The transfer of care from pediatric to adult healthcare has been associated with declines in health outcomes among adolescents and young adults with chronic conditions.* •* Psychiatric comorbidity may complicate the transition to adult healthcare.*	
**What is New:** • *Disease control, health-related quality of life, and experiences of care are good in most AYAs after the transfer of care to adult healthcare.* •* Mental well-being likely plays a more crucial and complex role in the transition to adult care than currently understood.*

**Supplementary Information:**

The online version contains supplementary material available at 10.1007/s00431-026-06791-z.

## Introduction

The transfer of care to adult healthcare is part of the broader transition process, marking the shift in care responsibility from pediatric to adult services [1]. At this stage, adolescents and young adults (AYAs) with chronic medical conditions should have developed necessary self-management skills to successfully navigate adult healthcare [1]. Physiological changes of puberty may explain why the disease control of some chronic medical conditions tends to worsen during adolescence [2, 3]. Low adherence to treatment and complications resulting from insufficient adherence are also common among AYAs [4]. The transfer of care is an acknowledged risk point when treatment outcomes may further deteriorate [5–7].

Most psychiatric conditions of adults emerge in adolescence [8]. Adolescents living with chronic medical conditions have an even higher risk of psychiatric disorders than their healthy peers [9, 10]. Psychiatric comorbidity can significantly influence the management and outcomes of chronic medical conditions [9, 11, 12]. A history of psychiatric diagnoses may be associated with an increased risk of an unsuccessful transition to adult healthcare [13]. Nonetheless, the specific effects of psychiatric comorbidity on treatment outcomes after the transfer of care are still poorly understood.

AYAs with chronic conditions often report lower health-related quality of life (HRQoL) compared to their healthy peers, and poorer HRQoL has been associated with suboptimal disease control [14, 15]. Experiences of care may significantly impact treatment adherence among AYAs, but care may not conform to established best practices for adolescent-friendly care [16]. However, few studies have examined how psychiatric comorbidity affects HRQoL or care experiences of AYAs with chronic conditions, especially during the transition of care.

We aimed to study changes in disease control across the transfer of care among AYAs living with chronic medical conditions. We also aimed to evaluate the associations of disease control and psychiatric comorbidity with HRQoL and experiences of care. We hypothesized that disease control would worsen after the transfer of care and that AYAs without psychiatric comorbidity would have better disease control, HRQoL, and experiences of care than AYAs with psychiatric diagnoses.

## Materials and methods

### Ethics

This study was approved by the Ethics Committee for Women’s and Children’s Health and Psychiatry at Helsinki University Hospital (HUS/1547/2017). All participants provided written informed consent. Fifteen adolescents were involved in the preparation of the study questionnaires [17]. This study conforms to the Declaration of Helsinki.

### Study design

This study is part of an international prospective cohort study, The Bridge [17]. In this study, we used only data collected from one study site—Helsinki University Hospital (HUS), Helsinki, Finland—due to a lack of clinical data from the other study site in Australia. We utilized data collected prior to the transfer of care (T0) and compared it with data collected 1 (T1) and 2 years (T2) after the transfer to adult healthcare.

### Participants

Participants were AYAs with various chronic medical conditions, treated at the New Children’s Hospital, from which they were recruited between September 2017 and August 2019. The recruitment process is detailed in the study protocol, which also outlines the inclusion criteria [17].

Participants were recruited from six subspecialties. The structure of transition services and the age at transfer varied across clinics (mean age at transfer, 17.3 years; ranging from 16.2 years in rheumatology to 19.0 years in AYAs with inflammatory bowel disease (IBD)) [18]. AYAs with diabetes mostly transfer to diabetes clinics operating within primary healthcare. AYAs with rheumatologic disease still requiring specialist care are transferred to a transition clinic at HUS, and AYAs in remission to primary healthcare. AYAs with IBD, cardiovascular disease, or solid organ transplant are transferred to respective adult clinics. AYAs with nephrological or neurological conditions are transferred either to adult specialist healthcare or to primary healthcare, depending on their condition and need for maintenance treatment.

### Clinical data

#### Disease control

We collected data to assess disease control from electronic medical records. Disease control was evaluated at three time points (T0, T1, T2) and divided into three categories: good control (good = 1), some evidence of concern (moderate = 2), and poor disease control (poor = 3) [17, 18]. Disease control was defined using the same criteria before and after transfer of care, except for AYAs with rheumatic diseases. In rheumatology, we use assessment tools designed for adults: the Disease Activity Score assessing 28 joints (DAS-28) for juvenile idiopathic arthritis (JIA), and the Ankylosing Spondylitis Disease Activity Score (ASDAS) or the Bath Ankylosing Spondylitis Disease Activity Index (BASDAI) for ankylosing spondylitis. The cutoff values for these measurements are presented in Supplementary Table 1 [19–21]. Changes in disease control were also assessed using mean HbA1c levels for diabetes and median fecal calprotectin (F-Calpro) levels for IBD. If disease control was not directly assessable using the predefined clinical criteria (16 AYAs at T1 and 12 at T2), two clinicians (MK and SK) independently assessed disease control based on available clinical data. At both time points, the initial assessments differed for five AYAs. For these cases, the evaluators jointly reviewed the data to reach consensus.

### Psychiatric diagnoses

Adolescent psychiatric diagnoses (ICD-10 Mental and behavioral disorders, F00–F99), covering ages 13–18 years, were extracted from electronic medical records. Please see the detailed data in Supplementary Table 2.

### Survey data

#### HRQoL measurements

HRQoL was assessed using two validated generic instruments for adolescents aged 12–18 to improve the reliability of the results.

##### Pediatric Quality of Life Inventory (PedsQL)

The PedsQL consists of 23 items covering physical, emotional, social, and school functioning over the past month. Responses are given on a 5-point Likert scale, ranging from “never a problem” to “almost always a problem.” Items are reverse-scored and transformed to a 0–100 scale, with higher scores indicating better HRQoL [22, 23]*.*

##### 16D

The 16D instrument is adapted from the 15D instrument, with items covering the 16 dimensions of health: mobility, vision, hearing, breathing, sleeping, eating, speech, excretion, discomfort and symptoms, depression, distress, mental function, vitality, physical appearance, school and hobbies, and relationships with friends [24, 25]. Each question has five response options. The 16D generates either a weighted profile with individual scores for each health dimension or a single index score representing overall HRQoL ranging from 0 (equivalent to death) to 1 (best possible HRQoL). The minimum important difference cross-sectionally and the minimum important change over time have been estimated to be ± 0.015 [26].

#### Experiences of care

Experiences of care were assessed using the Adolescent Friendly Hospital Survey, composed of eight items with the options “true,” “somewhat true,” “false,” or “I don’t know” (blank) [27]. A total score was calculated if at least seven items were answered, with missing data imputed as the means of other responses. Scores ranged from 8 to 24, with lower scores indicating a more positive experience.

### Missing data

The most common reasons for missing disease control data were transfer of follow-up to another district or primary care, discontinued follow-up, or missing scheduled visits. AYAs without disease control data were older at survey completion. AYAs who did not complete follow-up surveys were more often males. Supplementary Table 3 shows the sensitivity analysis of missing disease control and survey data.

### Statistical analysis

Statistical analyses were performed using IBM SPSS Statistics 29. All tests were two-tailed and *p* < 0.05 was considered significant. Categorical variables were described using frequencies and percentages. For continuous data, we reported means with standard deviations or medians with ranges depending on data distribution. We used Pearson’s chi-square test and Fisher’s exact test to assess the associations between categorical variables. The Wilcoxon signed rank test was used to compare disease control, HRQoL, and experiences of care at different time points. The Mann–Whitney *U* test was used to assess differences in HRQoL and total scores of experiences of care between groups with and without psychiatric comorbidity, and in sensitivity analyses between groups with missing data. The Kruskal–Wallis test with Bonferroni correction was used to examine differences in HRQoL and experiences of care between disease control groups. In most analyses, we combined AYAs with neurology, nephrology, and cardiovascular disease or organ transplant into a group labeled “others” due to the small number of participants in these subspecialties.

## Results

### Disease control

**Fig. 1:**
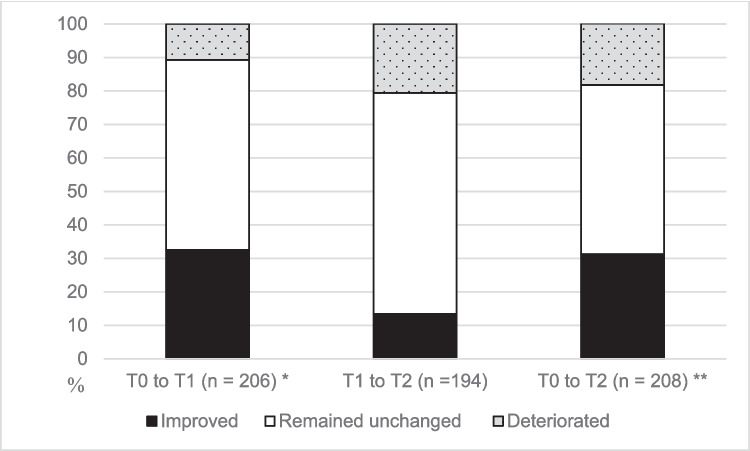
Changes in disease control of adolescents and young adults during the transfer of care. T0, before transfer of care; T1, 1 year after transfer of care; T2, 2 years after transfer of care; *p < 0.001; **p = 0.012 (Wilcoxon signed rank test)

Disease control classifications were available for 251 (99%) participants at T0, 206 (81%) at T1, and 208 (82%) at T2. Disease control remained unchanged for most adolescents (*n* = 117 (57%) 1 year and *n* = 105 (50%) 2 years after transfer of care) (Fig. 1). Disease control improved from T0 to T1 (*p* < 0.001) and from T0 to T2 (*p* = 0.011) in females and from T0 to T1 (*p* = 0.033) in males. No significant differences in disease control were observed between males and females. Disease control varied between diagnostic groups at all measurement points (*p* < 0.001 for T0, T1, and T2, respectively) (Table 1.). Disease control improved from T0 to T1 in AYAs with rheumatic disease (*p* < 0.001), but no statistically significant differences were observed in other diagnostic groups. Although the categorical disease control of AYAs with diabetes remained stable, after the transfer of care, the mean HbA1c levels increased to 64.6 mmol/mol (SD, 13.1) at T0, 67.3 mmol/mol at T1 (SD, 13.2; *p* = 0.031 for change), and 68.1 mmol/mol at T2 (SD, 16.5; *p* = 0.028 for change from T0), and the median F-Calpro levels decreased: 256 µg/g (range, 7–4342) at T0, 91 µg/g at T1 (range, 5–2247; *p* = 0.013 for change), and 114 µg/g at T2 (range, 5–1246; *p* = 0.064 for change from T0).

**Table 1:** Disease control of adolescents and young adults during the transfer of care according to diagnostic groups

Disease control, *n* (%)	All	Diabetes	Rheumatology	IBD	Others
Before transfer (T0)	251 (99)	91 (99)	66 (100)	45 (100)	49 (98)
Good	70 (28)	13 (14)	26 (39)	7 (16)	24 (49)
Moderate	105 (42)	45 (50)	24 (36)	23 (51)	13 (27)
Poor	76 (30)	33 (36)	16 (24)	15 (33)	12 (25)
1 year post-transfer (T1)	206 (81)	82 (89)	58 (88)	33 (73)	33 (66)
Good	94 (46)	10 (12)	52 (90)	13 (39)	19 (58)
Moderate	53 (26)	36 (44)	2 (3)	9 (27)	6 (18)
Poor	59 (29)	36 (44)	4 (7)	11 (33)	8 (24)
2 years post-transfer (T2)	208 (82)	83 (90)	59 (89)	30 (67)	36 (72)
Good	87 (42)	15 (18)	44 (75)	12 (40)	16 (44)
Moderate	56 (27)	31 (37)	11 (19)	6 (20)	8 (22)
Poor	65 (31)	37 (45)	4 (7)	12 (40)	12 (33)

Please note that percentages for each diagnostic group at each time point are calculated from the total study sample, whereas percentages of disease control categories are calculated within the diagnostic groups

*T0* before transfer of care, *T1* 1 year after transfer of care, *T2* 2 years after transfer of care, *IBD* inflammatory bowel disease, *Others* AYAs with neurology, nephrology, and cardiovascular disease or organ transplant

**Fig. 2:**
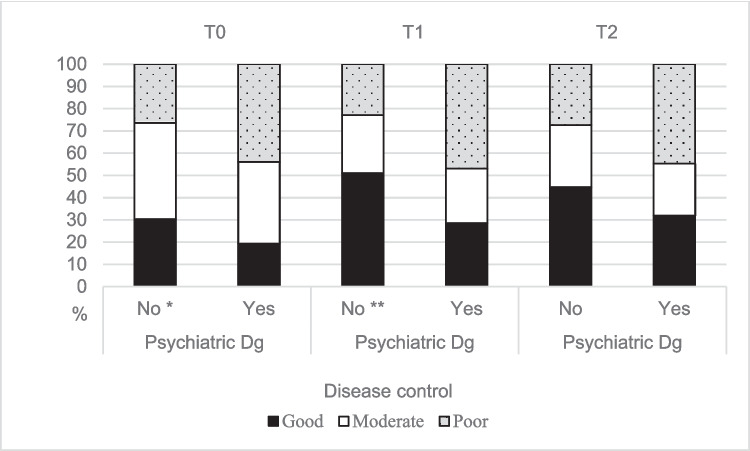
Disease control of adolescents and young adults with and without a comorbid psychiatric diagnosis during the transfer of care. Dg, diagnosis; T0, Before transfer of care; T1, 1 year after transfer of care; T2, 2 years after transfer of care; number of participants (with psychiatric Dg): T0 = 251 (57), T1 = 206 (49) and T2 = 208 (47); *p = 0.032; **p = 0.003; *p* = 0.072 at T2 (Pearson’s chi-square test)

AYAs with psychiatric comorbidity had worse disease control before transfer of care and 1 year after transfer of care than AYAs without psychiatric diagnosis (Fig. 2).

### HRQoL measurements

In all, 253 AYAs (52% females) participated in the surveys before the transfer of care, 199 (79%) 1 year after, and 172 (68%) 2 years after the transfer of care. PedsQL total scores and 16D single-index scores remained unchanged across the transfer of care. HRQoL showed no significant differences according to disease control except at T1, when AYAs with good and moderate disease controls had better 16D scores compared to those with poor control (mean single-index scores were 0.907, 0.906, and 0.858, and Bonferroni-adjusted* p* = 0.003 and 0.047, respectively).

AYAs with a comorbid psychiatric diagnosis had lower HRQoL scores than AYAs without psychiatric comorbidity in all PedsQL subdomains and in almost all 16D items (Table 2.). In 16D, AYAs with psychiatric diagnoses had lower scores at all three measurement points in questions regarding physical appearance, school and hobbies, and relationships with friends.

**Table 2:** The health-related quality of life scores of adolescents and young adults with and without psychiatric diagnoses during the transfer of care

No psych.dg/Psych.dg	Before transfer		1 year post-transfer		2 years post-transfer	
	*n* = 194	*n* = 58	*n* = 158	*n* = 41	*n* = 132	*n* = 40
**PedsQL mean scores**						
Total score	82.5	74.1**	82.2	72.1**	80.4	71.7*
Physical	86.2	81.6*	85.7	78.7*	85.3	76.7*
Emotional	76.9	65.0**	74.7	63.8*	71.2	64.6
Social	90.2	80.9**	91.2	79.9*	89.0	78.0*
School	74.7	64.2**	75.1	62.1**	73.1	64.4*
**16D mean scores**						
Single index score	0.920	0.854**	0.906	0.846*	0.903	0.855
Vitality	0.842	0.708**	0.794	0.698*	0.782	0.749
Vision	0.939	0.923	0.945	0.927	0.951	0.927
Breathing	0.952	0.904*	0.954	0.931	0.964	0.937
Distress	0.823	0.691**	0.762	0.712	0.748	0.690
Hearing	0.981	0.959*	0.974	0.952	0.983	0.971
Sleeping	0.840	0.735*	0.802	0.796	0.804	0.776
Eating	0.995	0.993	1.00	0.981*	0.997	1.00
Discomfort and symptoms	0.838	0.791	0.835	0.793	0.816	0.792
Speech	0.960	0.923	0.969	0.942	0.982	0.924*
Physical appearance	0.892	0.764**	0.881	0.713**	0.875	0.712**
School and hobbies	0.882	0.763**	0.866	0.771*	0.885	0.789*
Mobility	0.988	0.994	0.989	0.968	0.994	0.943*
Friends	0.971	0.876**	0.948	0.832**	0.939	0.840*
Mental function	0.955	0.922	0.941	0.879*	0.942	0.886
Excretion	0.925	0.861*	0.918	0.817*	0.876	0.898
Depression	0.860	0.739**	0.836	0.717*	0.809	0.739

*PedsQL* Pediatric Quality of Life Inventory, *No psych.dg* no psychiatric diagnoses, *Psych.dg* psychiatric diagnoses

*p < 0.05; **p < 0.001 (Mann–Whitney *U* test)

### Experiences of care

Experiences of care improved at T2 compared to T0 or T1 (*p* = 0.036 and 0.044). Disease control and psychiatric comorbidity showed no association with total experiences of care. When comparing individual questions of experiences of care, AYAs with psychiatric comorbidity felt less welcome in the hospital and less respected by the treating team before the transfer of care and were less comfortable asking questions about their health after the transfer of care (Table 3).

**Table 3:** Percentages of adolescents and young adults, with and without psychiatric diagnoses answering “true” or “false” to questions about their experiences of care during the transfer of care

During my last hospital visit…	Time point	True		False	
		No Psych.dg	Psych.dg	No Psych.dg	Psych.dg
**I felt welcome in the hospital**	T0**	94	76	0	0
	**T1**	80	78	2	0
	**T2**	83	90	2	0
**The hospital provided an age-appropriate environment for me**	**T0**	46	42	15	26
	**T1**	75	62	5	14
	**T2**	79	69	5	10
**I felt respected as a person by my treating team**	T0**	90	76	1	7
	**T1**	79	73	2	0
	**T2**	73	77	2	3
**I fully understood the health information provided**	**T0**	80	79	3	3
	**T1**	77	65	4	3
	**T2**	75	74	1	5
**I received enough information about my medical problems**^**a**^	**T0**	88	81	2	2
	**T1**	81	70	2	3
	**T2**	**-**	-	-	-
**I was sufficiently involved in decisions about my care and/or treatment**	**T0**	78	74	4	7
	T1*	76	54	5	8
	**T2**	74	69	6	6
**I felt comfortable to ask questions about my health and wellbeing**	**T0**	83	76	2	3
	T1*	77	57	2	11
	T2*	81	60	2	11
**I spent at least some of the consultation alone without my parents**	**T0**	78	80	12	10
	**T1**	93	84	3	5
	**T2**	94	95	4	0

Please note that percentages for the “somewhat true” responses are not shown to improve the clarity of the table. The number of responses to experience of care questions is as follows: T0 = 243–253, T1 = 168–171, and T2 = 164–170

*No psych.dg* no psychiatric diagnoses, *Psych.dg* psychiatric diagnoses, *T0* before transfer of care, *T1* 1 year after transfer of care, *T2* 2 years after transfer of care

^a^Missing data at T2 is due to the unintentional exclusion of this question from the questionnaire

*p < 0.05; **p < 0.001 between groups of adolescents and young adults with and without psychiatric diagnoses (Fisher’s exact test)

## Discussion

In this study, disease control was good or moderate in most of the AYAs and remained stable after transfer of care to adult care. The history of psychiatric comorbidity was associated with worse disease control and HRQoL both before and after the transfer, but disease control and psychiatric comorbidity showed no association with the scores of total experiences of care.

Contrary to our initial hypothesis, disease control remained stable after the transfer of care. When examining different diagnostic groups, disease control improved among AYAs with rheumatic diseases after the transfer of care, whereas no changes were found in other diagnostic groups. For AYAs with rheumatic diseases, disease control was assessed using the ten-joint juvenile arthritis disease activity score before transfer of care and DAS-28 afterward. However, DAS-28 may underestimate disease activity in AYAs with JIA [28], which could partly explain the observed improvement in disease control post-transfer. In AYAs with diabetes, the HbA1C levels rose after the transfer of care as seen in previous studies [29]. Instead, the F-Calpro levels of AYAs with IBD decreased. Transition recommendations have significantly improved the care of young people during the transition phase [29, 30], and these were already implemented in many of the subspecialties of our study—including a dedicated transition clinic for AYAs with rheumatic disease and joint appointments between pediatric and adult care providers for AYAs with clinically challenging IBD—potentially contributing to our positive findings.

The lack of statistically significant associations between disease control and HRQoL was an unexpected finding. In previous studies, worse disease control and self-reported health status have been associated with lower HRQoL [15, 31, 32]. In our study, HRQoL scores were already relatively high, and the sample size may have been insufficient to detect significant differences.

AYAs with chronic medical conditions are at increased risk of developing psychiatric disorders compared to their healthy peers [9, 10]. The transition from pediatric to adult care may further exacerbate vulnerability to psychiatric problems [11, 33, 34]. Psychiatric comorbidity represents an additional burden for AYAs and negatively impacts their disease control and overall well-being [35–38]. Psychiatric comorbidity also presents a challenge to clinicians preparing AYAs for transfer of care [39]. The tools used to assess transition readiness may not be fully applicable to AYAs with comorbid psychiatric diagnoses, and this population may require more tailored approaches, including focused discussions around the AYA’s own priorities and concerns regarding transition [40]. AYAs with psychiatric comorbidity could also benefit from a personalized care coordinator, such as an experienced social worker within the healthcare environment [41]. Research on transitioning to adult psychiatric services is limited. Only a small proportion of adolescents receiving psychiatric care transfer to adult mental health services, and a recent review highlights the need for stronger collaboration between child/adolescent and adult services [42, 43]. For AYAs with chronic medical conditions, a psychiatric condition may require a parallel transition of its own [44, 45].

Psychiatric comorbidity can complicate somatic conditions, leading to more complications, hospitalizations, and emergency visits [12, 41, 46]. In this study, worse disease control among AYAs with psychiatric comorbidity persisted across the transfer of care. Our study also supports the previous finding that AYAs with psychiatric comorbidity have consistently lower HRQoL throughout transition [45]. HRQoL was lower across all PedsQL domains among AYAs with psychiatric comorbidity compared to AYAs with only medical conditions. This suggests that psychiatric comorbidity has a broad impact on the life experience of adolescents with chronic medical conditions. Also, in 16D, AYAs with psychiatric comorbidity had worse mean scores, especially in questions regarding physical appearance, participation in schoolwork or hobbies, and social connections with peers. The close link between emotional well-being and participation and socializing is well known [47]. This highlights the importance of addressing both somatic and mental health as a unified whole while also considering the social environment in transition planning. Our results underline the need for integrated care that addresses both physical and mental health [38, 48–50].

In our study, AYAs reported positive experiences of care before and after transfer of care, which is in line with previous findings [16]. Neither disease control nor psychiatric comorbidity was associated with experiences of care before or after care transfer. This was an unexpected finding, as we hypothesized that suboptimal management of the somatic condition and concurrent psychiatric condition would negatively impact the experiences of care throughout the entire transition process. While disease control is undoubtedly important, many other factors probably influence AYAs’ experiences of care. It is encouraging to note that, despite the challenges of a chronic condition, AYAs can still feel valued and acknowledged in their care. In our previous research, self-reported transition-related anxiety was associated with negative care experiences after transfer of care [51]. In another study, the psychosocial HRQL of adolescents was a predictor of their satisfaction with care [52]. When comparing experiences of care in more detail, we found differences between AYAs with and without psychiatric diagnoses: AYAs with psychiatric comorbidity felt less welcome and less respected at the children’s hospital than AYAs without psychiatric comorbidity. This difference was not observed in adult care and may reflect a shift in clinical practice, where healthcare professionals increasingly engage with AYAs in a more holistic and individualized manner as they mature, incorporating mental well-being as an integral part of comprehensive care. After transferring to adult care, AYAs with psychiatric diagnoses felt less comfortable asking questions about their health and well-being. Actively encouraging AYAs’ participation during the consultation and in decision-making may help reduce anxiety and embarrassment, the most common barriers to question-asking [16].

A strength of our study is the good response rate at all time points, especially considering the often-low response rates found in the target population [16, 53, 54]. We were able to assess clinical outcomes for over 80% of AYAs transferred to adult healthcare. Our study included young people from various medical specialties, which suggests the results may be generalized to adolescents with chronic conditions more broadly. The follow-up period (median 2.6 years) was longer than in many previous studies of the transition phase. A limitation of this study is that only a few chronic conditions have specific disease control measures, such as HbA1c for diabetes, F-Calpro for IBD, or DAS-28 for rheumatoid arthritis. Therefore, our definitions of disease control for other diagnostic groups are mainly based on pre-established evaluation criteria developed through expert consultation [17]. However, we aimed to minimize subjectivity in the categorization process by having two authors independently assess disease control, with the final classification based on consensus. Despite that, the use of different disease control definitions for different conditions may have affected the results. The differences in assessment tools for AYAs with rheumatic diseases before and after the transfer of care also probably influenced the results. The use of three disease control groups may have been too rough to detect all changes in the clinical situation, as became evident when we used the exact HbA1c and F-Calpro values. The categories did, however, enable comparison of disease control across diagnostic groups.

In conclusion, the transition to adult healthcare does not necessarily lead to a deterioration of health outcomes, particularly when AYAs’ experiences of care are positive and the transition process is planned and adequately prepared. It is noteworthy that mental well-being may play an even more important and multidimensional role during the transition than it is currently understood. Further research is needed to explore how AYAs with chronic medical conditions and concurrent psychiatric comorbidity can be better supported during the transition to reduce disparities identified in this study.

## Supplementary Information

Below is the link to the electronic supplementary material.

ESM 1 (DOCX 20.0 KB)

## Data Availability

Data are available upon reasonable request. Some deidentified data are available from the first author upon reasonable request, taking into consideration data protection laws.

## Abbreviations

ASDAS: Ankylosing Spondylitis Disease Activity Score
AYAs: Adolescents and young adults
BASDAI: Bath Ankylosing Spondylitis Disease Activity Index
DAS-28: Disease Activity Score assessing 28 joints
F-Calpro: Fecal calprotectin
HRQoL: Health-related quality of life
HUS: Helsinki University Hospital
IBD: Inflammatory bowel disease
JIA: Juvenile idiopathic arthritis
PedsQL: Pediatric Quality of Life Inventory
T0: At children’s hospital before transfer of care
T1: One year after transfer of care
T2: Two years after transfer of care
