# Supplementary material for: Disease control, psychiatric comorbidity, health-related quality of life, and experiences of care during transition to adult healthcare: a single-center prospective study
Source: Eur J Pediatr. 2026 Mar 11;185(4):174. doi: 10.1007/s00431-026-06791-z (PMC12979283; doi:10.1007/s00431-026-06791-z)
Supplement: Supplementary file 1 — (DOCX 20.0 KB) [file 431_2026_6791_MOESM1_ESM.docx]

**Disease control, psychiatric comorbidity, health-related quality of life and experiences of care during transition to adult healthcare: a single-centre prospective study**

Mira Kallio ^a,b^, Anna Alanen ^b,c^, Kaija-Leena Kolho ^a,b^, Heikki Relas ^d^, Silja Kosola ^b^

Affiliations: Department of Pediatrics, University of Helsinki and Helsinki University Hospital, Helsinki, Finland; b New Children`s Hospital, Pediatric Research Center, University of Helsinki and Helsinki University Hospital, Helsinki, Finland; c Department of Nursing Science, University of Turku, Turku, Finland; d Inflammation Center, University of Helsinki and Helsinki University Hospital, Helsinki, Finland

**Corresponding author:** Mira Kallio, mira.kallio@hus.fi

**Journal:** European Journal of Pediatrics

**Supplementary table 1** The cutoff values used for assessing disease control in rheumatic conditions after transfer of care to adult healthcare

| **Disease control** | **DAS-28** | **ASDAS ₐ** | **BASDAI ₐ** |
| --- | --- | --- | --- |
| **Good** | < 2.6 | < 1.3 | < 1.9 |
| **Moderate** | 2.6 - 3.2 | 1.3 - 2.1 | 1.9 – 3.5 |
| **Poor** | > 3.2 | > 2.1 | > 3.5 |

DAS-28 = the Disease Activity Score Assessing 28 joints, ASDAS= Ankylosing Spondylitis Disease Activity Score, BASDAI = Bath Ankylosing Spondylitis Disease Activity Index

ₐ The presence of active iritis automatically results in classification of disease control as poor.

**Supplementary table 2** The psychiatric diagnoses of adolescents. Diagnoses were classified according to ICD-10 three-character categories, each including all corresponding four-character subcategories (e.g., F32 includes F32.0–F32.9).

| **Diagnosis** | **Number of diagnoses** |
| --- | --- |
| F43 Reactions to severe stress and adjustment disorders | 25 |
| F32 Depressive episode | 18 |
| F41 Other anxiety disorders | 11 |
| F93 Emotional disorders with onset specific to childhood | 7 |
| F40 Phobic anxiety disorders | 4 |
| F42 Obsessive–compulsive disorder | 4 |
| F90 Hyperkinetic disorders | 4 |
| F54 Psychological or behavioral factors associated with disorders or diseases classified elsewhere | 3 |
| F59 Unspecified behavioral syndromes associated with physiological disturbances and physical factors | 3 |
| F84 Pervasive developmental disorders | 3 |
| Other diagnoses ₐ | 21 |

ₐ the “other diagnoses” group comprised diagnoses with low frequency (one or two cases), such as F50 Eating disorders and F94 Disorders of social functioning with onset specific to childhood and adolescence.

**Supplementary table 3** Comparison of missing data groups of AYAs for disease control and survey completions at time points T1 and T2

|  | **PedsQL** | **16D** | **Experience of care** | **Age at survey completion** | **Gender** |
| --- | --- | --- | --- | --- | --- |
| **Disease control**  **T1** | T0: p=0.60  T1: p=0.72  T2: p=0.62 | T0: p=0.21  T1: p=0.72  T2: p=0.77 | T0: p=0.78  T1: p=0.32  **T2: p=0.031*** | T0: p=0.15  **T1: p=0.003***  **T2: p=0.005*** | p=0.18  Pearson Chi-Square |
| **Disease control**  **T2** | T0: p=0.33  T1: p=0.96  T2: p=0.99 | T0: p=0.23  T1: p=0.89  T2: p=0.98 | T0: p=0.98  T1: p=0.99  T2: p=0.051 | **T0: p=0.003****  **T1: p<0.001****  **T2: p<0.001**** | p=0.80  Pearson Chi-Square |
| **Survey**  **T1** | T0: p=0.20  T1: -  T2: 0.35 | T0: p=0.34  T1: -  T2: p=0.26 | T0: p=0.69  T1: -  T2: p=0.28 | T0: p=0.34  T1: -  **T2: p=0.01***** | **p=0.013*****  Pearson Chi-Square |
| **Survey**  **T2** | T0: p=0.31  T1: p=0.38  T2: - | **T0: p=0.03******  T1: p=0.30  T2: - | T0: p=0.60  T1: p=0.55  T2: - | T0: p=0.06  T1: p=0.57  T2: - | **p=0.002******  Pearson Chi-Square |

AYAs = Adolescents and young adults, PedsQL = Pediatric Quality of Life Inventory**,** T0 = Before transfer of care, T1= One year after transfer of care, T2= two years after transfer of care.

p < 0.05 is considered significant, Mann-Whitney U test (All comparisons have been made using the data available at each respective time point)

* AYAs whose disease control was undetermined at T1 had worse experience of care at T2 (mean scores 10.6 vs 9.7) and were older during survey completion at T1 and T2 (mean ages T1 18.9 vs 18.2 years and T2 20.5 vs 19.7 years)

** AYAs whose disease control was undetermined at T2 were older during survey completion at T0, T1 and T2 (mean ages T0 17.8 vs 17.0 years, T1 19.1 vs 18.2 years and T2 20.8 vs 19.6 years)

*** AYAs who did not complete the survey at T1 were older during survey completion at T2 (mean ages 21.0 vs 19.7 years) and were more often males (61% vs 37%)

**** AYAs who did not complete the survey at T2 had better 16D scores at T1 (mean scores 0.91 vs 0.89) and were more often males (61% vs 38%)
